# 

**Published:** 1917-05

**Authors:** 


### ANNOUNCEMENTS

---

AMERICAN MEDICAL ASSOCIATION.—New York City, June 4 to 8. (Clinical—4 and 5.) The American Medical Association will hold its anual meeting in New York City, June 4 to 8, 1917. In all probability this will be the greatest meeting of medical men ever held in this country. The Committee on Arrangements consists of Drs. Wendell C. Phillips, Floyd M. Crandall, and Alexander Lambert. Headquarters at 17 West 43d st., New York City, to which all communications should be addressed. Monday and Tuesday, June 4 and 5, will be devoted to a Clinical Congress. All departments of medicine will be represented, each department to have its own series of clinics, occurring in different parts of the city.

The great number of clinics, their scope and scientific value, will be unprecedented. The program is in active preparation, soon to appear in full in the *Journal* of the A. M. A.

Between seven and ten thousand physicians are expected to attend this congress, and it is hoped that dental practitioners throughout the country will utilize this opportunity to become associate fellows of the Section on Stomatology, and thus be entitled to attend these clinics as well as the regular meeting of the association on June 6-8. The following is from a letter of the General Secretary:

"Dentists holding the degree of D.D.S. who are members of state or local dental societies and who are not eligible to regular membership, may be elected to associate fellowship by the House of Delegates of the American Medical Association when a formal application for such associate fellowship has been approved by the Section on Stomatology. In other words, when a dentist holding the required degree makes formal application for associate fellowship, accompanying this application with a certificate from the secretary of his state or local dental society, which certificate certifies to his being a member in good standing in that body, this application and the attached certificate is forwarded to the secretary of the Section on Stomatology for the approval of that section. The application is then in form for presentation to the House of Delegates for the election or rejection of the applicant.

We shall be glad to transmit any such applications, or they may be sent direct to the secretary of the Section on Stomatology, Dr. Eugene S. Talbot, 31 N. State st., Chicago.''

Headquarters of the Section on Stomatology will be at the Hotel Astor, Broadway and 45th st.

No more impressive opportunity for the fraternization of dental and medical men has ever been presented in the history of the profession. Every dentist who reads this announcement should enroll immediately in the Section on Stomatology (annual dues $5, which entitles him to the weekly *Journal* of the A. M. A.), and thus place himself in direct touch with the greatest happenings in medical science.

The Clinical Committee of the Section on Stomatology is as follows: Henry W. Gillett, Arthur L. Swift, Wm. D. Tracy, Henry S. Dunning, Arthur H. Merritt, Wm. B. Dunning, Chairman, 140 West 57th st., New York City.

---

THE FOUR STATES POSTGRADUATE MEETING, which is to hold its meeting June 4 to 7 in New Orleans, promises to be an unusually attractive meeting.

If the large number of essayists and clinicians announced materialize, it certainly will be worth while going miles to attend. A large number of the most active men in the profession will make up the programme, and the topics are all of exceeding interest. We don't see how any one who can attend can afford not to do so.

---

CHANGE OF DATE. The Connecticut State Dental Society will hold its annual meeting in New London, June 28 to 30 instead of 14 to 16 as formely announced. The meetings will be held in the beautiful Griswold Hotel, and a fine programme is prepared.

---

IOWA STATE BOARD OF EXAMINERS will hold its meeting for registration, June 4 in Iowa City.

---

MICHIGAN BOARD OF EXAMINERS will hold examinations in Ann Arbor, June 18.

---

ILLINOIS STATE BOARD will meet in Northwestern Dental College, Chicago, June 15.

---

THE FORSYTH DENTAL INFIRMARY FOR CHILDREN, 140 The Fenway, Boston. Permanent staff appointments. A competitive examination of graduates in dentistry (of less than three years standing) for appointments to positions on the permanent staff for full and one-half time service will be held early in June at the infirmary.

Appointments will be made for one or two years as follows:

Full time service requiring operating five and one-half days week at a salary of $1,000 a year.

One-half time service requiring operating six half-days a week, either forenoon or afternoon, at a salary of $400 a year.

These appointments will be made subject to satisfying the requirements of the Massachusetts State Board of Registration in Dentistry and to ''qualifying'' in the practical work of the clinics during one month's trial.

Members of this staff will be entitled to the advantages of reports and clinics by experts in the various branches of dentistry from different parts of the world in addition to the numerous regular clinics and lectures.

Operators after serving three months are eligible, by qualifying, for appointments in the special clinics where postgraduate work is given.

The operators on this staff have the advantage of the clinics and lectures of the Postgraduate School of Orthodontia.

The infirmary clinics provide unusual advantages in the various departments of the institution where operative dentistry, orthodontia, nose and throat and oral surgery, extracting, novocaine, technic, radiography, pathological diagnosis and research work are continually carried on.

The average number of cases treated daily is more than 450 in all departments.

All material and necessary operating instruments will be furnished; up-to-date apparatus including electric engines, sterile instrument trays, fountain cuspidors, compressed air, and the modern operating room type of lavatories are available for use.

A diploma of service will be issued by the trustees to each member of this staff who has completed this term of service in a satisfactory manner.

Applications for the above positions should be made not later than May 15.

Information and the date of the examination will be furnished to those interested.

Harold DeW. Cross, D.M.D. Director, 140 The Fenway, Boston, Mass.

